# A 17.8–20.2 GHz Compact Vector-Sum Phase Shifter in 130 nm SiGe BiCMOS Technology for LEO Gateways Receivers

**DOI:** 10.3390/mi14061184

**Published:** 2023-05-31

**Authors:** Javier del Pino, Sunil L. Khemchandani, Mario San-Miguel-Montesdeoca, Sergio Mateos-Angulo, Daniel Mayor-Duarte, Jose Luis Saiz-Perez, David Galante-Sempere

**Affiliations:** 1 Institute for Applied Microelectronics (IUMA), Universidad de Las Palmas de Gran Canaria, 35017 Las Palmas de Gran Canaria, Spain; sunil@iuma.ulpgc.es (S.L.K.); dgalante@iuma.ulpgc.es (D.G.-S.); 2 Wireless Innovative MMIC (WIMMIC), 35017 Las Palmas de Gran Canaria, Spain; mario.sanmiguel@wimmic.com (M.S.-M.-M.); sergio.mateos@wimmic.com (S.M.-A.); daniel.mayor@wimmic.com (D.M.-D.); jose.saiz@wimmic.com (J.L.S.-P.)

**Keywords:** phased array, SiGe, vector modulator (VM), vector-sum phase shifter (VSPS)

## Abstract

This paper presents a novel and compact vector modulator (VM) architecture implemented in 130 nm SiGe BiCMOS technology. The design is suitable for use in receive phased arrays for the gateways of major low Earth orbit (LEO) constellations that operate in the 17.8 to 20.2 GHz frequency range. The proposed architecture uses four variable gain amplifiers (VGA) that are active at any given time and are switched to generate the four quadrants. Compared to conventional architectures, this structure is more compact and produces double the output amplitude. The design offers 6-bit phase control for 360°, and the total root mean square (RMS) phase and gain errors are 2.36° and 1.46 dB, respectively. The design occupies an area of 1309.4 μm × 1783.8 μm (including pads).

## 1. Introduction

The need for fast data rate and widely available mobile communications systems is driving the adoption of satellite communication (SATCOM) technologies. Companies such as SpaceX, Telesat, OneWeb, and Amazon are developing large low Earth orbit (LEO) mega-constellations that require ground terminals capable of tracking multiple satellites [1]. Unlike conventional satellite systems, this type of system requires a large number of gateway antennas located near or co-located with internet peering points. Phased arrays are becoming a popular solution for this purpose, offering improved directivity and the ability to electronically steer the beam and operate with multiple satellites simultaneously. This results in an improved signal-to-noise ratio (SNR) and increased directional resilience to interference [2].

**Figure 1:**
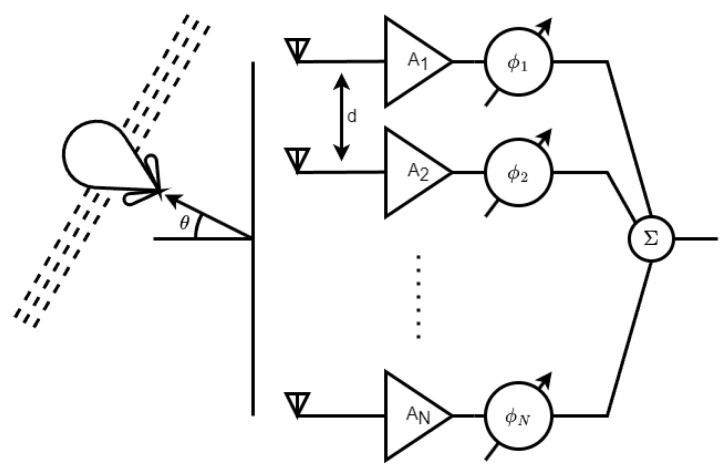
Phased array block diagram.

As illustrated in Figure 1, a phased array consists of a number of antennas driven by a common source and arranged to direct individual phases in a particular direction while suppressing them in others. When operating at high frequencies, the separation between the elements is reduced, resulting in a need for minimal physical space. To effectively integrate the elements in these configurations, it is essential to utilize low-cost, low-power consumption circuits. One possible solution is to use microwave integrated circuits (MMICs) in the array electronics, as they provide the required performance in a compact area.

The key components for directing and focusing the beam in a phased array are phase shifters. These electronic devices change the phase of the elements in the antenna array to direct the main radiation beam. Phase shifters are typically implemented using vector-sum phase shifters (VSPS) or vector modulators (VM). This paper presents a novel and compact VM architecture implemented in 130 nm SiGe BiCMOS technology, suitable for use in receive phased arrays for the gateways of major LEO constellations operating in the 17.8 to 20.2 GHz frequency range. The proposed architecture uses four variable gain amplifiers (VGA) that are active at any given time and are switched to generate the four quadrants. Compared to conventional architectures, this structure is more compact and produces double the output amplitude.

The organization of this paper is as follows. Section 2 describes the state-of-the-art and the proposed vector modulator. Section 3 presents the circuit design of the proposed vector-sum phase shifter. Section 4 shows the measurement results and Section 5 concludes this paper.

## 2. State-of-the-Art and Proposed Vector Modulator

**Figure 2:**
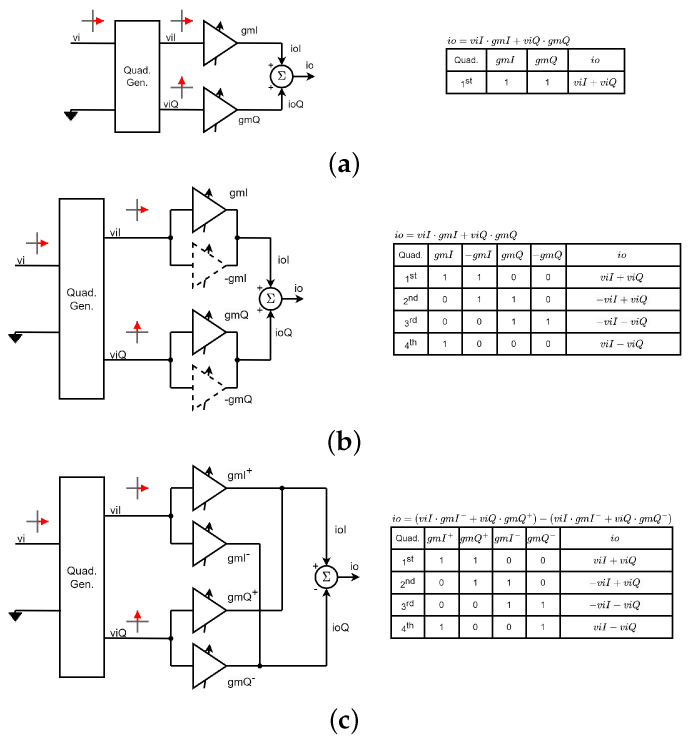
Single-ended vector-sum phase shifters: (**a**) 90° based on two non-inverting amplifiers; (**b**) 360° based on two inverting and two non-inverting amplifiers; (**c**) 360° based on four non-inverting amplifiers with crossed outputs.

As shown in Figure 2a, vector modulators create phase variations by combining in-phase (viI) and quadrature (viQ) versions of the input signal with different weights. The phase of the output signal can be controlled by varying the in-phase and quadrature gains, gmI and gmQ. However, changing these gains also affects the output amplitude, so the gains must be carefully chosen to maintain a constant output amplitude. This architecture only allows for 90° phase changes. To achieve 360° phase shifts, two additional inverting amplifiers are typically added, as shown in Figure 2b. In each quadrant, only one *I* and one *Q* amplifier are active. To avoid using inverting and non-inverting amplifiers, the architecture with crossed outputs shown in Figure 2c is often used [3,4,5].

**Figure 3:**
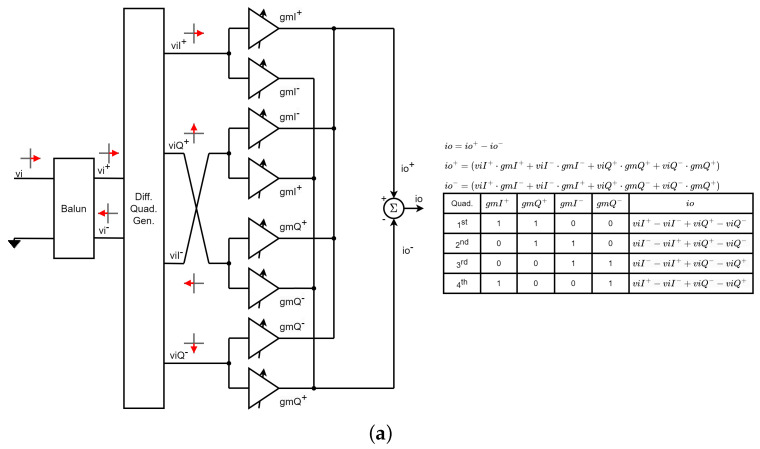

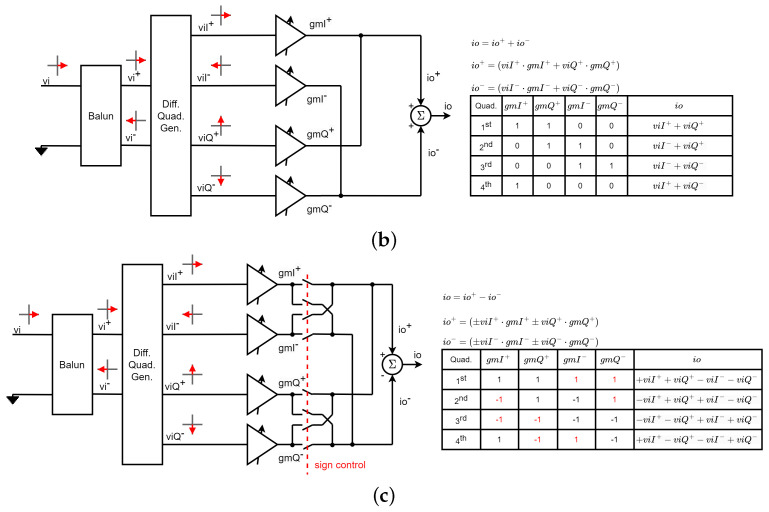
Differential vector-sum phase shifters: (**a**) 360° based on a double Gilbert-cell; (**b**) 360° based on a single Gilbert-cell and an adder at the output; (**c**) 360° based on a single Gilbert-cell, a subtracter at the output, and four switches to control the quadrant (proposed architecture).

The architectures in Figure 2a–c use single-ended signals. However, it is generally preferred to work with differential signals to improve the common-mode rejection ratio. Figure 3a shows a commonly used differential VSPS based on a double Gilbert-cell structure. First, a balun converts the single-ended input signal to differential mode and then a differential quadrature generator generates the in-phase and quadrature differential versions of the input signal. Two Gilbert cell-type variable gain amplifiers (VGAs) are then used to create the final vector by adding the current-domain signals at the output nodes with appropriate gains to give the desired phase. This architecture requires at least four digital to analog converters (DACs), one for each VGA that is active at any given time [6,7]. However, for each quadrant, only two different gains need to be controlled, so by switching the DAC that controls each gain, only two DACs are needed. This technique is commonly used to reduce the number of DACs needed [8,9,10,11,12,13,14,15,16,17,18,19,20,21,22,23,24,25,26,27,28,29,30,31].

To reduce the number of VGAs and, therefore, the area and consumption, some authors have proposed the architecture shown in Figure 3b [32,33,34]. The particularity of this scheme is that it uses an adder element at the output instead of a subtractor. With this architecture, the four quadrants are covered, but the amplitude of the output signal is half compared to the architecture in Figure 3a. If an output subtractor is used instead of an adder, it would not be possible to cover all four quadrants. In this work, we propose a modification of this scheme, which consists of using a subtracter at the output and inserting the switches shown in Figure 3c. In this way, if we allow the four VGAs to be active at the same time, the four quadrants are covered and, in addition, the output amplitude would be the same as in Figure 3a.

**Figure 4:**
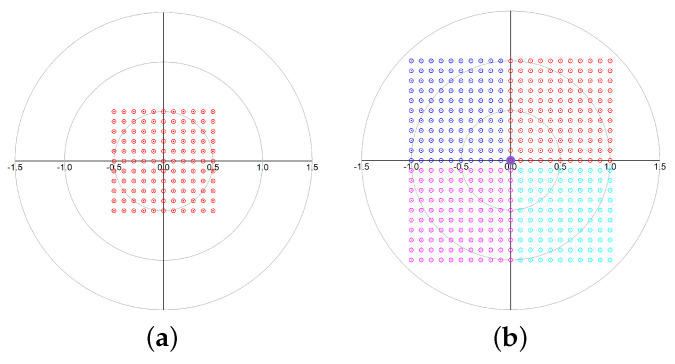
Constellations: (**a**) generated from Figure 3b circuit; (**b**) generated from Figure 3c circuit.

To further analyze the performance of the proposed architecture, Figure 4 shows the constellations generated from the circuits of Figure 3b,c when the four gains $gmI^{+}$, $gmI^{-}$, $gmQ^{+}$, and $gmQ^{-}$ are swept between 0 and 1 in steps of 0.2. As can be seen, Figure 4a shows a single constellation covering the four quadrants while Figure 4b shows four constellations, one for each of the quadrants, depending on the position of the quadrant control switches. Based on these results, it can be stated that, with this topology, the gain can be doubled and a higher phase resolution can be achieved for the same number of steps, i.e., bit resolution.

## 3. Circuit Design

**Figure 5:**
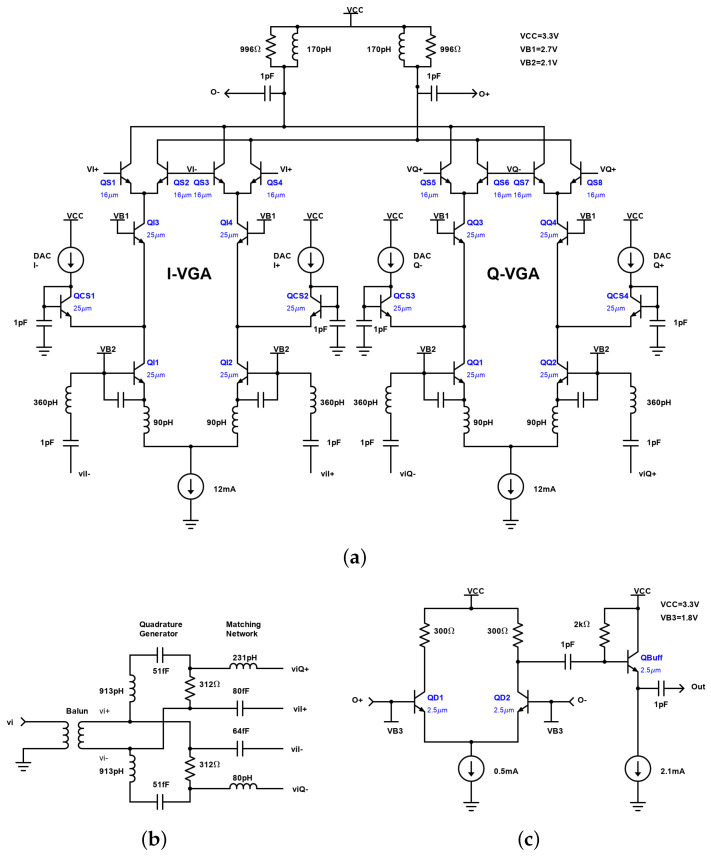
Implementation of the architecture proposed in Figure 3c based on bipolar transistors: (**a**) schematic of the VGAs; (**b**) input balun, differential I/Q generator, and matching network; (**c**) output subtractor based on a differential amplifier and buffer.

For demonstration, a full 360° phase-shifting range VSPS employing the proposed architecture is implemented in the 130 nm SiGe BiCMOS GlobalFoundries process. Figure 5 shows an implementation of the architecture proposed in Figure 3c based on bipolar transistors. First, a balun converts the single-ended input signal into differential mode, and then a differential quadrature generator provides the in-phase and quadrature differential versions of the input signal. To improve input matching and noise figure (NF), an LC matching network is inserted between the quadrature generator and the input of the VGAs. The VGAs in this circuit are built using cascode amplifiers (QI1–QI4 and QQ1–QQ4), with the addition of four pairs of transistors (QS1–QS8) at the top that are selectively activated to select the quadrant. These transistors function as switches and are controlled by the voltages applied to their bases: $VI^{+}$, $VI^{-}$, $VQ^{+}$, and $VQ^{-}$. Within each quadrant, the phase is chosen using the current steering technique to control the gain of each of the branches (QCS1–QCS4). This is accomplished by acting on the DACs associated with each of the branches: $DACI^{+}$, $DACI^{-}$, $DACQ^{+}$, and $DACQ^{-}$. The advantage of using this technique is that the tail current is always the same and, therefore, the transconductance (gm) of the input transistors remains constant for all phase values. This results in a constant input impedance, so the input matching remains unchanged.

**Figure 6:**
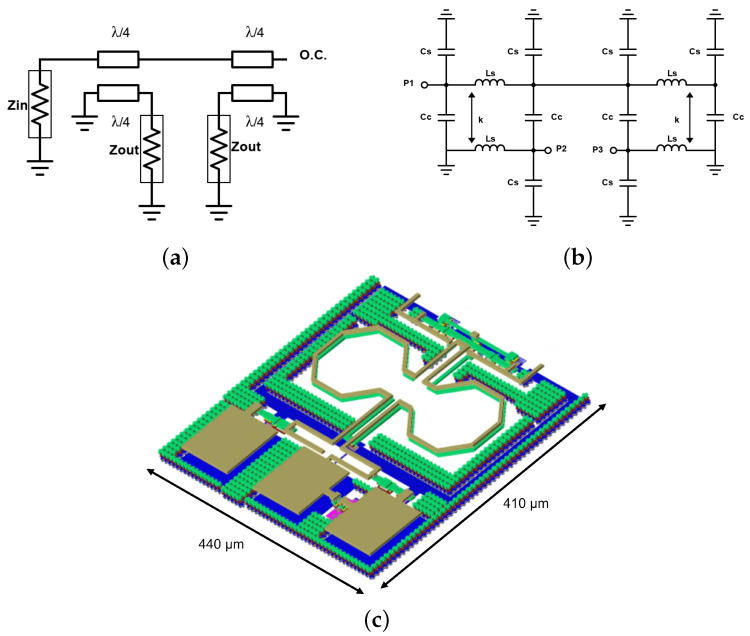
Marchand balun: (**a**) distributed model, (**b**) lumped model, and (**c**) 3-D momentum model.

In the circuit design process, one of the most challenging components is the input balun, which allows the conversion of single-ended signals to differential signals at the input. Various types of passive baluns, including transformer baluns, rat-race baluns, and Marchand baluns, have been utilized in MMIC designs. Among these options, the Marchand balun stands out as the preferred choice for millimeter-wave designs due to its simplicity and wideband performance [35]. As depicted in Figure 6a, a conventional distributed-element Marchand balun consists of two quarter-wavelength coupled lines. This configuration ensures balanced signals across the load impedances ($Z_{out}$) at the two output ports when driven by an unbalanced signal at the input port ($Z_{in}$). Typically, the load impedances differ from the driving impedance ($Z_{0}$) at the input port. The primary limitation of distributed-element Marchand baluns arises from their size when applied to the relatively low frequencies used in LEO constellation receiver gateways (17.8 to 20.2 GHz). This limitation is attributed to the significant size of the required λ/4-lines, making it challenging to achieve compact designs. To address this size limitation, we employ a lumped-component Marchand balun based on the structure proposed by [35], as shown in Figure 6b. The λ/4-lines are replaced with π-type lumped component equivalents, and the inductances ($L_{s}$) are coupled together to introduce additional mutual inductance and reduce the overall area requirement. The required inductance ($L_{s}$), coupling factor (*k*), shunt capacitance ($C_{s}$), and coupled capacitance ($C_{c}$) are determined using Equations (1)–(4), where the characteristic odd-mode and even-mode impedances ($Z_{oo}$, $Z_{oe}$) and transformation ratio (*C*) are given by Equations (5)–(7), [35]:

(1) $$L_{s}=\frac{Z_{oe}+Z_{oo}}{2\omega }$$

(2) $$k=\frac{Z_{oe}-Z_{oo}}{2\omega L_{s}}$$

(3) $$C_{s}=\frac{1}{\omega Z_{oe}}$$

(4) $$C_{c}=\frac{1}{2\omega Z_{oo}}-0.5C_{s}$$

(5) $$Z_{oo}=Z_{0}\sqrt{\frac{1-C}{1+C}}$$

(6) $$Z_{oe}=Z_{0}\frac{Z_{0}}{Z_{oo}}$$

(7) $$C=\frac{1}{\sqrt{2\frac{Z_{L}}{Z_{0}}+1}}$$

At the intended carrier frequency of 19 GHz, these equations yield the following component values: $L_{s}$ = 513 pH, k=0.577, $C_{s}$ = 86.7 fF, and $C_{c}=118.5$ fF. The capacitors can be directly obtained from the process design kit (PDK) models, with MIM-type high-density capacitors of 87 fF and 118 fF used for $C_{s}$ and $C_{c}$, respectively. These capacitors offer a compact area and exhibit the lowest process tolerance among the available options in the PDK components. However, the same level of control is not achievable for the required inductances ($L_{s}$) and the desired coupling factor (*k*), necessitating several electromagnetic (EM) simulations to achieve the desired combination of these values. Since the implementation of inductors with more than one turn results in a self-resonant frequency (SRF) too close to the intended carrier frequency, a one-turn inductor is used. A 3-D Momentum model of the designed Marchand balun, including the input ground-signal-ground (GSG) pads, is shown in Figure 6c. The Marchand balun results in a total chip size of 440 μm × 410 μm.

## 4. Measurement Results

**Figure 7:**
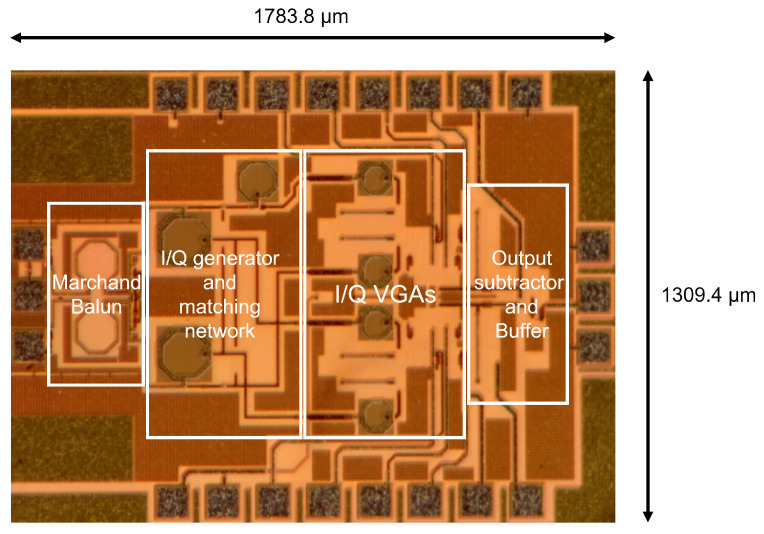
Die photograph of the fabricated chip.

The vector modulator described in this paper was fabricated using a 130 nm SiGe BiCMOS GlobalFoundries process (GF-8XP). Figure 7 shows a chip micro-photograph of the modulator, which occupies an area of 1309.4 μm × 1783.8 μm (with pads).

**Figure 8:**
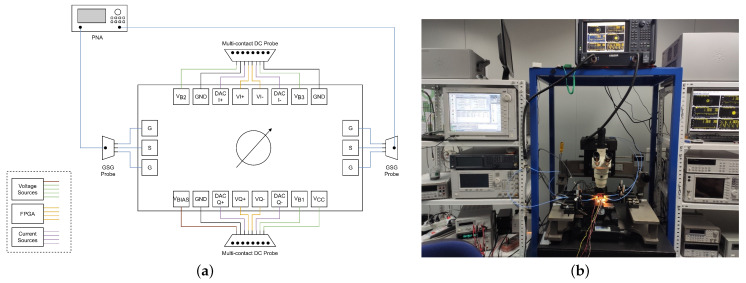
Measurement setup: (**a**) blocks diagram and (**b**) photograph.

Figure 8 shows a blocks diagram and a photograph of the measurement setup. The chip was characterized on-wafer using a Cascade SUMMIT 9000 probe station and a Keysight N5225B PNA Network Analyzer. Ground-signal-ground (GSG) probes were employed at the input and output ports. Additionally, multi-contact DC probes were utilized for setting the biasing voltages and currents. The biasing currents were generated using current mirrors biased with the voltage source $V_{BIAS}$. DC voltage sources were employed to generate $V_{CC}$, $V_{B1}$, $V_{B2}$, and $V_{B3}$, while the quadrant selection voltages ($VI^{+}$, $VI^{-}$, $VQ^{+}$, and $VQ^{-}$) were generated using an external FPGA. Finally, the Keysight B1500A Semiconductor Device Parameter Analyzer Source Measure Units (SMU) configured as current sources were used to generate the currents for controlling the phase shifting ($DACI^{+}$, $DACI^{-}$, $DACQ^{+}$, and $DACQ^{-}$). The modulator consumes 25 mA under 3.3 V including the output subtractor.

**Figure 9:**
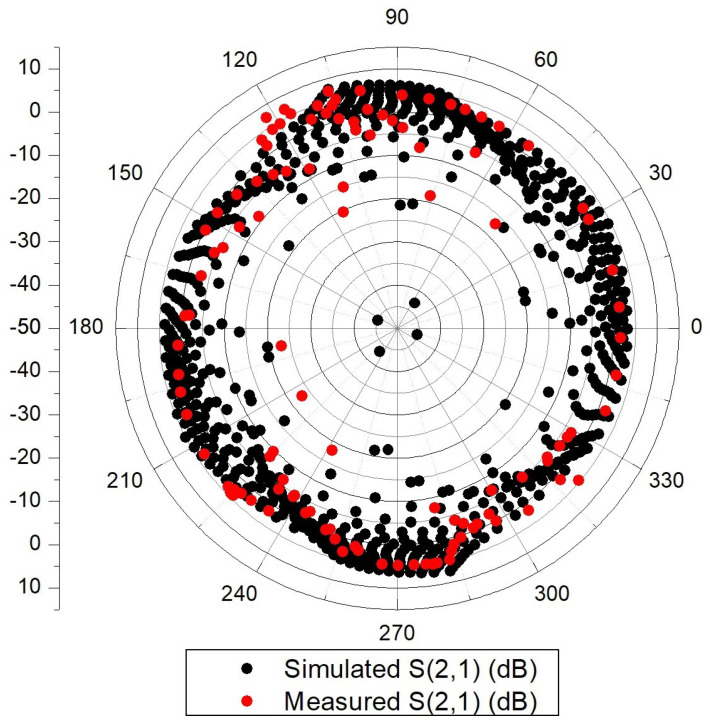
Simulated and measured phase shifting at 19 GHz (polar plot).

Figure 9 displays the simulation and measurement results for the S21 parameter at 19 GHz as a function of phase shift. In the simulation, 8-bit DACs with a minimum LSB-bit current step of 35 μA were used to generate steering currents that control the gain of each branch. For a 360° phase range, the simulated S21 ranges from 7.5 to −45 dB. By carefully selecting the DAC currents, it was possible to achieve the desired phase jumps with a constant amplitude. For instance, for a constant amplitude of 0 dB and 5.625° phase jumps (i.e., 64 states or 6-bit resolution), the RMS phase and gain errors are only 2.36° and 1.46 dB at 19 GHz.

**Figure 10:**
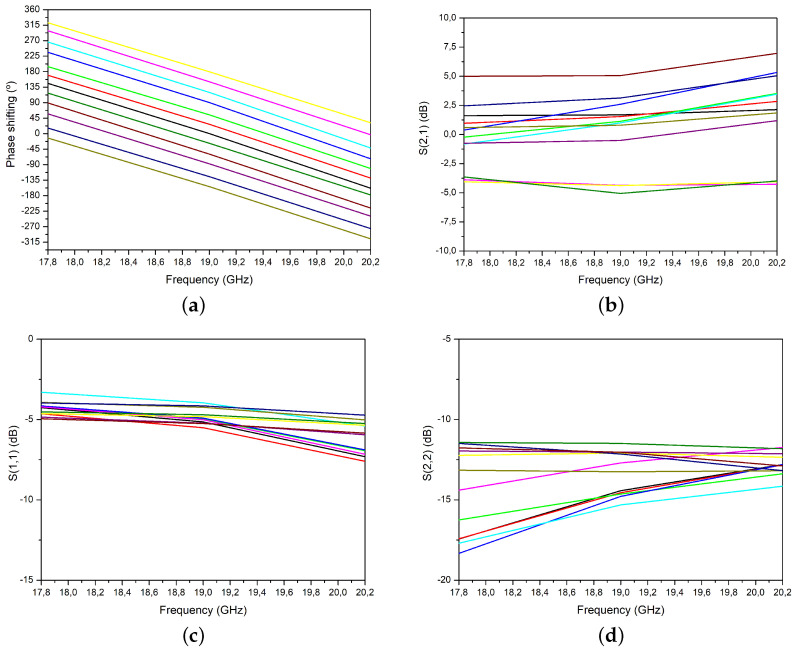
Measured frequency response: (**a**) phase-shift, (**b**) S21, (**c**) S11, and (**d**) S22.

The vector modulator was tested in 112 different states covering the 360° circle. Figure 10a,b depict the measured phase shift and gain as a function of frequency from 17.8 to 20.2 GHz for phase angles ranging from 0° to 360° with steps of 30°. Although the measured cases do not provide enough data to determine the value of the RMS phase and gain errors, the graph demonstrates that the vector modulator can generate phases from 0° to 360° over the entire 2.4 GHz band. As shown, the modulator exhibits a relatively flat gain across the frequency band with minimal deviation. Furthermore, the phase shift shows a linear trend, indicating that the circuit can achieve accurate phase modulation over the entire frequency range.

Figure 10c,d present the measured S11 and S22 parameters of the vector modulator for the selected states. As can be seen, the output is well matched (S22 < −10 dB) in all cases. The input matching, however, is slightly worse due to insertion losses incurred by the input balun. In this paper, we propose the use of a lumped-component Marchand balun topology for its potential to reduce the required area at the operating frequencies [35]. This approach combines distributed elements with lumped components so that, instead of using quarter-wavelength transmission lines, we substitute them with pi-type lumped component equivalents. By coupling the inductors together, we are able to increase mutual inductance and further minimize the area needed. This approach not only allows for a reduction in size, but also results in a more compact and efficient design. However, the input matching is negatively affected.

In Table 1, a comparison is presented between this work and other phase shifters that operate at similar frequencies and with comparable phase resolutions. The results indicate that the proposed phase shifter achieved a highly competitive performance compared to the other works. This is attributed to the innovative technique employed in the design, which enabled the phase shifter to attain a figure of merit (FoM) that is surpassed only by [11]. This phase shifter has a much higher gain due to the inclusion of an active balun that dominates the gain. Despite this, our circuit displays the second-best RMS phase error and performs similarly to other works with respect to RMS gain error, even though no additional amplifiers were included in the design.

## 5. Conclusions

This paper describes the design of a vector modulator circuit for use in receive phased arrays of major LEO constellations operating in the 17.8 to 20.2 GHz frequency range. The modulator uses a compact architecture composed of four VGAs that are active at any given time and are switched to generate the four quadrants. The circuit was fabricated using a 130 nm SiGe BiCMOS process and occupies an area of 1309.4 μm × 1783.8 μm (with pads). The simulation and measurement results demonstrate that the vector modulator is capable of generating every phase from 0° to 360° with a 6-bit phase resolution over the 2.4 GHz band.

## Figures and Tables

**Table 1:** Comparison of state-of-art active VSPSs.

	This Work	[33]	[19]	[11]	[36]	[37]	[10]
**Tech.**	130 nm SiGe	250 nm SiGe	65 nm CMOS	180 nm SiGe	180 nm SiGe	130 nm CMOS	130 nm CMOS
**Freq. (GHz)**	17.8–20.2	8–12	15–38	6–18	8–12	12–18	6–18
**No. of bits**	6	6	6	5	5	6	4
**Gain (dB)**	0	−2.5 *	−1.7	19.5 *	1.75 *	1 *	−0.2
**RMS phase** **error (°)**	2.36	2	3.5	5.6	4.6	4	10
**RMS gain** **error (dB)**	1.46	6.4	1	1.1	0.6	0.9	1.7
**Pdc (mW)**	82	110	19.2	61.7	73.9	37.5	8.7
**Core area** **(mm^2^)**	1.23	1.65	0.16	0.27	0.6	0.24	0.14
**FoM** ${}^{†}$	1.82	0.56	1.26	7.43	1.42	1.53	0.32

* An additional amplifier and/or active balun is included. ^†^ $\mathrm{FoM}=\frac{{\mathrm{Gain}}_{\mathrm{lin}.}\;\times \;\mathrm{No}.\mathrm{bits}}{\theta_{\mathrm{RMSerror}}({}^{o})\;\times \;{\mathrm{Gain}}_{\mathrm{RMSerror},\mathrm{lin}}}$

## Abbreviations

The following abbreviations are used in this manuscript:

DACs	Digital to Analog Converter
FoM	Figure of Merit
LEO	Low Earth Orbit
MMIC	Monolithic Microwave Integrated Circuit
NF	Noise figure
RMS	Root Mean Square
SATCOM	Satellite Communication
SNR	Signal-to-Noise Ratio
VGA	Variable Gain Amplifier
VM	Vector Modulator
VSPS	Vector-Sum Phase Shifter
